# Multifactorial Competition and Resistance in a Two-Species Bacterial System

**DOI:** 10.1371/journal.pgen.1005715

**Published:** 2015-12-08

**Authors:** Anupama Khare, Saeed Tavazoie

**Affiliations:** 1 Department of Systems Biology, Columbia University, New York, New York, United States of America; 2 Department of Biochemistry and Molecular Biology, Columbia University, New York, New York, United States of America; University of Michigan, UNITED STATES

## Abstract

Microorganisms exist almost exclusively in interactive multispecies communities, but genetic determinants of the fitness of interacting bacteria, and accessible adaptive pathways, remain uncharacterized. Here, using a two-species system, we studied the antagonism of *Pseudomonas aeruginosa* against *Escherichia coli*. Our unbiased genome-scale approach enabled us to identify multiple factors that explained the entire antagonism observed. We discovered both forms of ecological competition–sequestration of iron led to exploitative competition, while phenazine exposure engendered interference competition. We used laboratory evolution to discover adaptive evolutionary trajectories in our system. In the presence of *P*. *aeruginosa* toxins, *E*. *coli* populations showed parallel molecular evolution and adaptive convergence at the gene-level. The multiple resistance pathways discovered provide novel insights into mechanisms of toxin entry and activity. Our study reveals the molecular complexity of a simple two-species interaction, an important first-step in the application of systems biology to detailed molecular dissection of interactions within native microbiomes.

## Author Summary

Bacteria commonly exist in nature as part of large multispecies communities, and their behavior is affected by the surrounding species via secreted molecules or physical contact. Such interactions are poorly understood, and the pathways that actually affect bacterial growth and behavior in any multispecies system have rarely been studied. In this study, we show that the opportunistic pathogen *Pseudomonas aeruginosa* inhibits the growth of the commensal *Escherichia coli*, and we use unbiased genome-scale methods to identify the mediators. We find that *P*. *aeruginosa* iron-chelating molecules and redox-active phenazines account for all of the *E*. *coli* growth inhibition seen in our system. We also evolve *E*. *coli* in the presence of the *P*. *aeruginosa* antimicrobials and identify multiple pathways that lead to resistance, gaining novel insights into the mechanism of action of these antimicrobial molecules. Thus, our study demonstrates the complexity of even simple two-species bacterial systems and lays down a framework for studying such interactions at the molecular level.

## Introduction

Microorganisms are typically found in complex communities such as those in the soil, aquatic environments, and the microbiome [1], and interactions between microbial species can critically impact their survival and evolutionary trajectories [1, 2]. Current knowledge suggests that competition plays an important role in interspecies microbial interactions [3, 4]. This includes both exploitative competition, where species compete for limited nutrients, as well as interference competition, where species directly antagonize each other [5]. However, such ecological processes are understudied and poorly characterized in microbial systems [2].

Previous studies have identified molecules produced by bacteria that may affect the behavior or fitness of other species. Such molecules could be beneficial to the target species [6], but a wide variety of them have been shown to be antagonistic in nature [7–9]. In most cases, such studies have looked at a single molecule or class of molecules, and the potential effects these could have on exogenous bacteria. However, the entire breadth of interactions that actually determines fitness in a specific multispecies system has rarely been identified and characterized at the molecular level.

As communities are established, bacteria evolve in response to the biotic and abiotic challenges present. Although adaptation to various physicochemical stresses has been widely studied (for example [10]), the mechanisms that underlie adaptation to interspecies competition remain largely unknown. The immediate cellular effect of toxic exoproducts on target bacteria has been described for some interactions, but how target populations can evolve upon such exposure to combat the antimicrobials has not been studied.

Here we systematically dissect interactions in a two-species bacterial system containing *P*. *aeruginosa* and *E*. *coli*. *P*. *aeruginosa*, an opportunistic pathogen, is frequently found in multi-species infections [11, 12] and is capable of interacting with other microorganisms via a variety of antimicrobial molecules [8]. The other interacting partner, *E*. *coli*, is a commensal and the best studied bacterial species, which we utilized as a model target organism. Our genome-scale analyses of this two-species bacterial system identified both interference and exploitative competition, mediated by multiple molecules in the antagonistic species, which explained all of the observed competition. We also discovered several diverse genetic determinants of resistance in the target species, gaining insights into the properties of adaptive trajectories in the face of interspecies competition.

## Results

### *P*. *aeruginosa s*ecretes molecule(s) that inhibit *E*. *coli* growth

**Fig 1:**
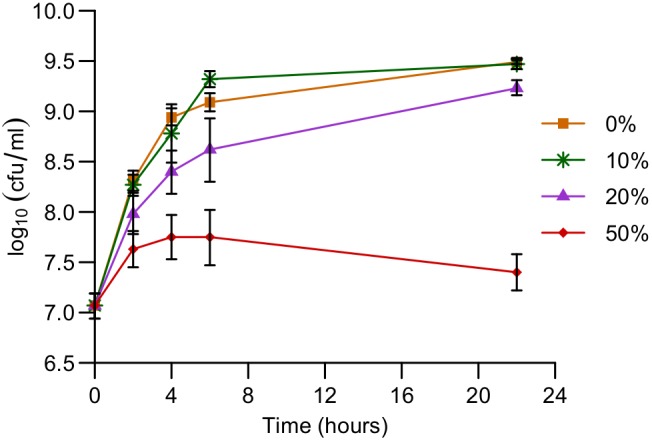
*P*. *aeruginosa* inhibits the growth of *E*. *coli*. *E*. *coli* cells were grown in the presence of different % (v/v) of WT *P*. *aeruginosa* spent media, and the cell density was determined at several time-points. Data are the means from 5 replicates. Error bars represent the standard deviation. The growth in the presence of 20% and 50% spent media even at 4h was significantly different from that under control conditions (*q* < 0.01), but the growth in the presence of 10% spent media was not (*q* > 0.3), as determined by a one-sided Mann-Whitney *U* test followed by the Benjamini-Hochberg procedure for multiple testing correction.

We studied a bi-species system containing *P*. *aeruginosa* and *E*. *coli* in planktonic culture (using media conditions in which they have very similar growth rates). We tested whether any interactions are seen between these species, by measuring the relative fitness of wild-type (WT) *E*. *coli* and *P*. *aeruginosa* in direct competition with each other. *E*. *coli* cells were found to have a relative fitness of 0.2 (± 0.09), which is significantly less than 1, revealing that *P*. *aeruginosa* was inhibiting *E*. *coli* growth. Further, *E*. *coli* cells showed substantial reduction in growth upon exposure to *P*. *aeruginosa* spent media for a few hours (Fig 1), indicating that, at least part of the *P*. *aeruginosa* antagonism was mediated by secreted molecules. Each of the conditions in this experiment had the same volume of fresh media (50%) to enable comparison across conditions, and the remainder was made up of the appropriate volume of spent media added to the media salts base. *E*. *coli* spent media did not have a significant effect on *P*. *aeruginosa* growth (S1 Fig).

### Iron-limitation by *P*. *aeruginosa* siderophores limits *E*. *coli* growth

**Fig 2:**
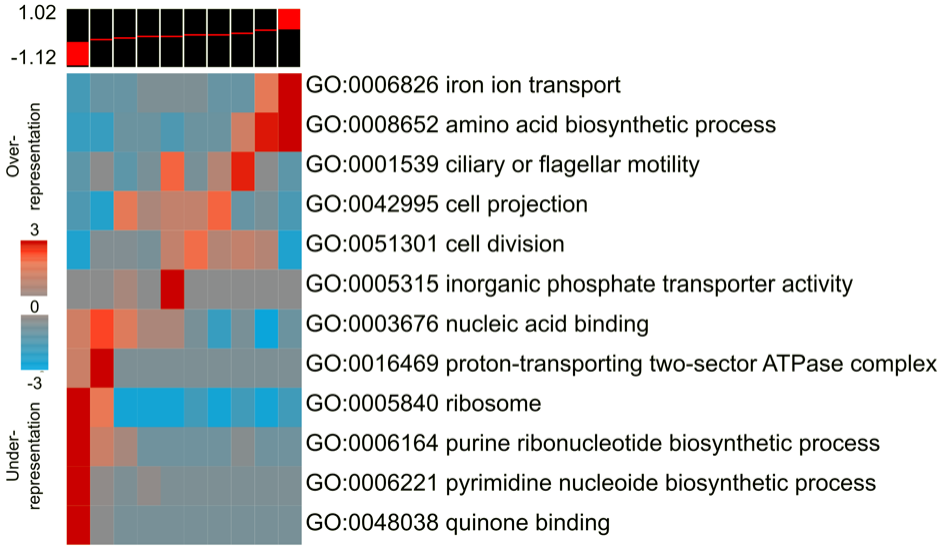
*P*. *aeruginosa* spent media induces iron transport pathways. *E*. *coli* cells were grown in the presence of 20% (v/v) *P*. *aeruginosa* spent media for 20min, and the transcriptional response was measured compared to unexposed cells. The genes were ordered by their fold-induction and divided into 10 equal sized bins, which are represented in the 10 columns. The range of log_10_ (fold-change) is shown on the top left of the heat-map. The global change in gene expression was analyzed using iPAGE [13], which identifies the functional GO categories significantly enriched and depleted across the bins, as depicted in the heat map. The colors show the significance, with red representing the negative of log_10_ of the over-representation *p*-values, and blue representing the log_10_ of the under-representation *p*-values.

We determined the global transcriptional response of *E*. *coli* to *P*. *aeruginosa* spent media, and identified the Gene Ontology (GO) annotations enriched (and depleted) across the full range of change in gene expression, using iPAGE, a mutual-information based pathway analysis tool [13]. One of the most strongly induced pathways was iron transport (Fig 2). *P*. *aeruginosa* is known to secrete two siderophores, pyoverdine and pyochelin, that chelate iron and transport it inside the cell [14]. Although these molecules are thought to have evolved primarily for iron acquisition by the producer, they may also limit iron-availability for other microbial species within the community. *P*. *aeruginosa* spent media also induced several amino acid biosynthesis pathways, while repressing genes involved in core cellular processes such as ribosomal translation, nucleotide biosynthesis, ATP synthesis, and the electron transport chain (Fig 2). These changes are similar to those produced during the stringent response, which is known to be induced by iron starvation [15, 16]. Additionally, genes involved in ciliary or flagellar motility are also upregulated, which may represent an adaptive response to enable migration away from competition or to niches with higher iron availability.

Mass spectrometry (both MALDI and ESI) on whole spent media from WT *P*. *aeruginosa* revealed a major component that had an *m/z* of 1335 Daltons (S2 Fig), which matches the molecular mass of pyoverdine [17, 18]. We also fractionated the spent media by reverse-phase HPLC using an acetonitrile-water gradient, and tested the fractions for growth-inhibitory activity against *E*. *coli*. While some bioactive fractions showed a complex mass-spectrometry profile making it difficult to identify the active component, the main component in one of the active fractions also had an *m/z* of 1335 Daltons (S3 Fig).

**Fig 3:**
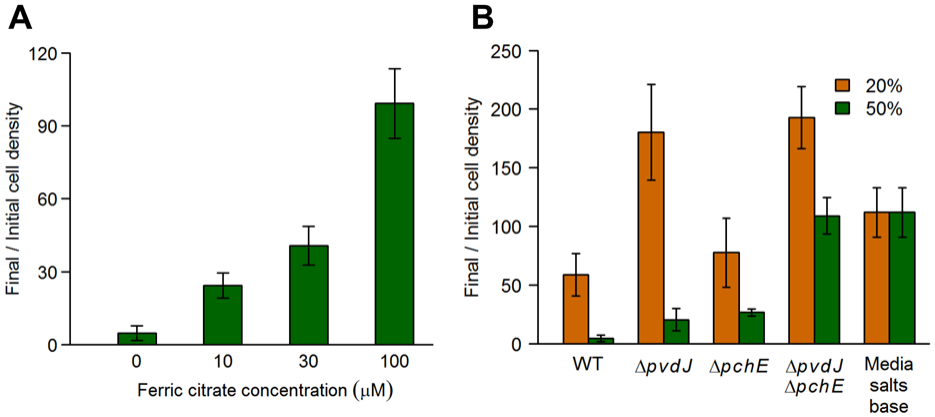
Iron-limitation by *P*. *aeruginosa* siderophores inhibits *E*. *coli* growth. *E*. *coli* cells were grown in the presence of *P*. *aeruginosa* spent media and the cell density was determined before and after 16 hours of growth. Data are the means from at least 5 replicates. Error bars represent standard deviation. **(A)** *E*. *coli* cells were grown in the presence of 50% (v/v) *P*. *aeruginosa* spent media supplemented with increasing levels of ferric citrate. Data from each concentration were significantly different from the preceding concentration (*q* < 0.005) as determined by a one-sided Mann-Whitney *U* test followed by the Benjamini-Hochberg procedure for multiple testing correction. **(B)** *E*. *coli* cells were grown in the presence of 20% or 50% (v/v) spent media from various *P*. *aeruginosa* wild-type and mutant strains. All mutant and control data shown (except for Δ*pchE* at 20%) were significantly different from WT (*q* < 0.005) as determined by a one-sided Mann-Whitney *U* test followed by the Benjamini-Hochberg procedure for multiple testing correction (this correction included the mutant strains showed in Figs 5 and 7A). Spent media from the Δ*pvdJ* and the Δ*pvdJ* Δ*pchE* mutants supported more growth than the media salts base control, likely due to the presence of unused nutrients or *P*. *aeruginosa* signaling molecules in the spent media.

**Fig 4:**
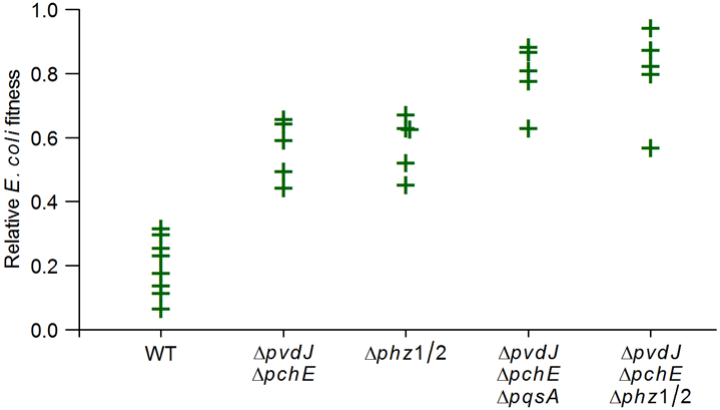
*Pseudomonas* siderophores and phenazines reduce fitness of *E*. *coli* in direct competition with *P*. *aeruginosa*. *E*. *coli* cells were grown in direct competition with *P*. *aeruginosa* WT and mutant strains, and the fitness of both species was measured over a period of 20 hours. All mutant data were significantly different from WT (*q* < 0.001) as determined by a one-sided Mann-Whitney *U* test followed by the Benjamini-Hochberg procedure for multiple testing correction. The fitness of the mutant strains in competition with *E*. *coli* was similar to that of WT *P*. *aeruginosa*, supporting the idea that the siderophores and phenazines actively decrease *E*. *coli* fitness, rather than increasing *P*. *aeruginosa* fitness under our conditions.

Consistent with the iron-sequestration mechanism of competition, the addition of surplus iron partially alleviated the growth inhibition of *E*. *coli* by *P*. *aeruginosa* spent media in a concentration-dependent manner (Fig 3A). Iron supplementation also caused a marginal 1.4-fold increase in growth under control conditions (S4 Fig), with the effect saturating at 10μM ferric citrate supplementation. This mild iron limitation in the growth media does not account for the up to 27-fold increase in growth caused by iron supplementation in the presence of *P*. *aeruginosa* spent media, indicating that the spent media was causing the significant iron limitation seen. Further, deletion of genes encoding key enzymes in the pyoverdine (*pvdJ*) and pyochelin (*pchE*) biosynthetic pathways, singly and in combination, caused significantly lower growth inhibition of *E*. *coli* than the WT (Fig 3B). *E*. *coli* also had higher relative fitness in competition with a *P*. *aeruginosa* siderophore double mutant, compared to WT (Fig 4), confirming that iron-limitation by the *P*. *aeruginosa* siderophores pyoverdine and pyochelin engenders exploitative competition and inhibits the growth of *E*. *coli*, in our system.

### PQS-induced phenazine molecules inhibit *E*. *coli* growth

**Fig 5:**
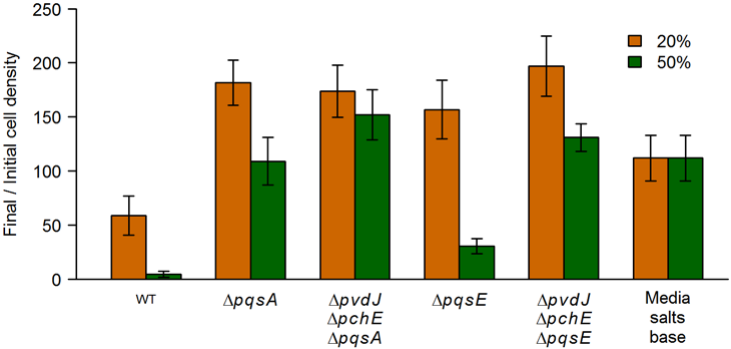
The PQS pathway response in *P*. *aeruginosa* is important for *E*. *coli* growth inhibition. *E*. *coli* cells were grown in the presence of 20% or 50% (v/v) spent media from either WT *P*. *aeruginosa* or various PQS pathway mutants, and the cell density was determined before and after 16 hours of growth. Data are the means from at least 5 replicates. Error bars represent standard deviation. All mutant and control data shown were significantly different from WT (*q* < 0.005) as determined by a one-sided Mann-Whitney *U* test followed by the Benjamini-Hochberg procedure for multiple testing correction (this correction included the mutant strains showed in Figs 3B and 7A).

One of the major *P*. *aeruginosa* quorum sensing molecules, PQS (*Pseudomonas* Quinolone Signal), is also known to chelate ferric ions [19, 20]. We tested a deletion mutant for a gene encoding a key enzyme in the PQS biosynthesis pathway (*pqsA*). Spent media from the Δ*pqsA* mutant as well as a Δ*pvdJ* Δ*pchE* Δ*pqsA* mutant caused significantly lower growth inhibition of *E*. *coli* compared to the WT (Fig 5), and *E*. *coli* had higher relative fitness in competition with these mutants as compared to WT *P*. *aeruginosa* (Fig 4) demonstrating that the PQS pathway is also involved in the antagonism.

PQS not only chelates iron, but also induces a wide range of virulence factors such as hydrogen cyanide, rhamnolipids, lectin, and phenazines [21], via the PQS-response protein PqsE. A Δ*pqsE* mutant was deficient in inhibiting *E*. *coli* growth (Fig 5), indicating that the PQS pathway molecules inhibited *E*. *coli* growth indirectly, likely through the expression of one or more virulence factors.

**Fig 6:**
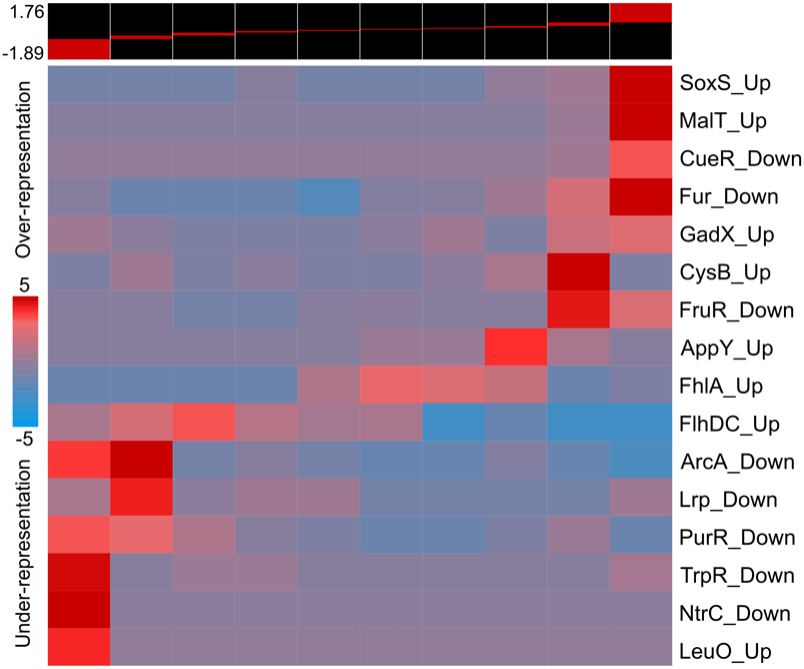
*P*. *aeruginosa* spent media upregulates the *soxS*-response in *E*. *coli*. *E*. *coli* cells were grown in the presence of 45% (v/v) WT *P*. *aeruginosa* spent media supplemented with 100μM ferric citrate, and the transcriptional response was measured compared to unexposed cells. The genes were ordered by their fold-induction, and divided into 10 equal sized bins, represented in the 10 columns. The range of the log_10_(fold-change) is shown on the top left. The global change in gene expression was analyzed using the ‘transcription factor regulon’ module in iPAGE [22], which identifies the transcription factor regulons enriched and depleted across the bins, as shown in the heat map. The colors show the significance of the enrichment, with red representing the negative of log_10_ of the over-representation *p*-values, and blue representing the log_10_ of the under-representation *p*-values.

To identify these factor(s), we determined the transcriptional response of *E*. *coli* to WT *P*. *aeruginosa* spent media supplemented with ferric citrate (to eliminate the effect of iron-limitation), and analyzed the results using iPAGE [13]. The ‘transcription factor regulon’ module in iPAGE [22] identified the SoxRS regulon as being enriched in the upregulated genes (Fig 6). Interestingly, *soxS* has recently been shown to be upregulated in *E*. *coli* in response to several biotic stresses including a *Vibrio cholera* strain known to kill *E*. *coli*, the P1*vir* bacteriophage, and the antimicrobial peptide Polymyxin B, likely to protect against reactive oxygen species generated due to these stresses [23]. The *P*. *aeruginosa* secondary metabolite pyocyanin (the terminal phenazine molecule) is known to upregulate the *soxS*-response in *P*. *aeruginosa* [24]. Further, phenazines are known to be PQS-induced, and to have antimicrobial properties, likely due to the production of reactive oxygen species or inhibition of bacterial respiration [25–27]. We thus hypothesized that the phenazine pathway was responsible for the PQS-mediated growth inhibition of *E*. *coli*.

**Fig 7:**
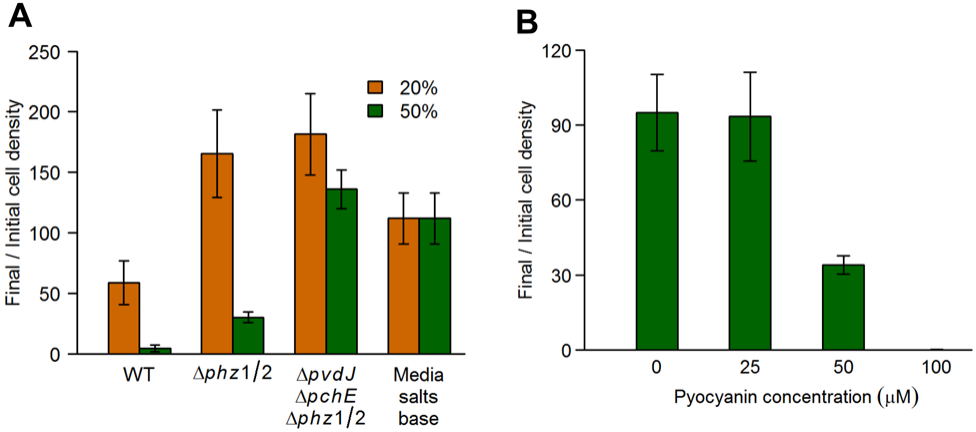
*P*. *aeruginosa* secreted phenazines inhibit *E*. *coli* growth. *E*. *coli* cells were grown in the presence of *P*. *aeruginosa* spent media or pyocyanin, and the cell density was determined before and after 16 hours of growth. Data are the means from at least 5 replicates. Error bars represent standard deviation. **(A)** *E*. *coli* cells were grown in the presence of 20% or 50% (v/v) spent media from *P*. *aeruginosa* wild-type and phenazine mutant strains. All mutant and control data shown were significantly different from WT (*q* < 0.005) as determined by a one-sided Mann-Whitney *U* test followed by the Benjamini-Hochberg procedure for multiple testing correction (this correction included the mutant strains showed in Figs 3B and 5). **(B)** *E*. *coli* cells were grown in the presence of increasing concentrations of pyocyanin. The 50μM and 100μM data were significantly different from the immediately lower concentration (25μM and 50μM, respectively) as determined by a one-sided Mann-Whitney *U* test followed by the Benjamini-Hochberg procedure for multiple testing correction (*q* < 0.01), but the 25μM data was not significantly different from the 0μM data (*q* > 0.5).

Spent media from a mutant lacking both copies of the phenazine biosynthesis operon (Δ*phz*1/2), as well as a Δ*pvdJ* Δ*pchE* Δ*phz*1/2 mutant, showed significantly reduced *E*. *coli* growth inhibition (Fig 7A), and *E*. *coli* had higher relative fitness in competition with these mutants (Fig 4), compared to the WT. Further, pyocyanin, the terminal phenazine molecule, caused concentration-dependent growth inhibition of *E*. *coli* (Fig 7B), confirming that phenazine molecules directly inhibit *E*. *coli* growth via interference competition. The concentration of pyocyanin used in our experiments (25–100μM) covers the range of measured pyocyanin concentrations in the growth media of PA14 (30–60μM) in both minimal media as well as LB [24].

Importantly, *E*. *coli* had a relative fitness of almost 1 in competition with *P*. *aeruginosa* strains deficient for both siderophores and phenazines (Fig 4), demonstrating that these molecules account for the entirety of the measurable *P*. *aeruginosa* antagonism against *E*. *coli*. Thus, using unbiased genomic level approaches, we have identified pathways by which *P*. *aeruginosa* inhibits the growth of *E*. *coli* in the conditions under study, via both exploitative and interference competition. *P*. *aeruginosa* limits iron availability in the environment, thereby shutting down most core cellular processes in *E*. *coli* cells. The phenazine molecules further limit *E*. *coli* growth possibly by inhibiting cellular respiration [25, 26], and inducing the production of reactive oxygen radicals in the *E*. *coli* cells that are still able to respire aerobically [27].

### *E*. *coli* can evolve resistance to *P*. *aeruginosa* antimicrobials through multiple pathways

Despite identification of individual molecules that can mediate competitive interspecies interactions, the mechanisms of adaptation to such competition and the attributes of the adaptive solutions have remained largely unstudied. To identify pathways by which *E*. *coli* can resist *P*. *aeruginosa* antimicrobials, we carried out laboratory evolution of *E*. *coli* in the presence of either WT spent media, Δ*pvdJ* spent media, or pyocyanin. We exposed WT *E*. *coli* to increasing concentrations of the spent media or pyocyanin, and performed 7–19 daily transfers into the selective media (the transfers were stopped when the cultures did not show significantly improved growth under the selective condition for two consecutive days). We then carried out whole genome sequencing on 2 or 3 clones each from 2 or 3 populations evolved under each condition. All the evolved clones had between 1–9 mutations, with recurring mutations in *mprA* in the spent-media selected clones, and in *fpr* and *ompC* in the pyocyanin-selected clones (S1 Table).

**Fig 8:**
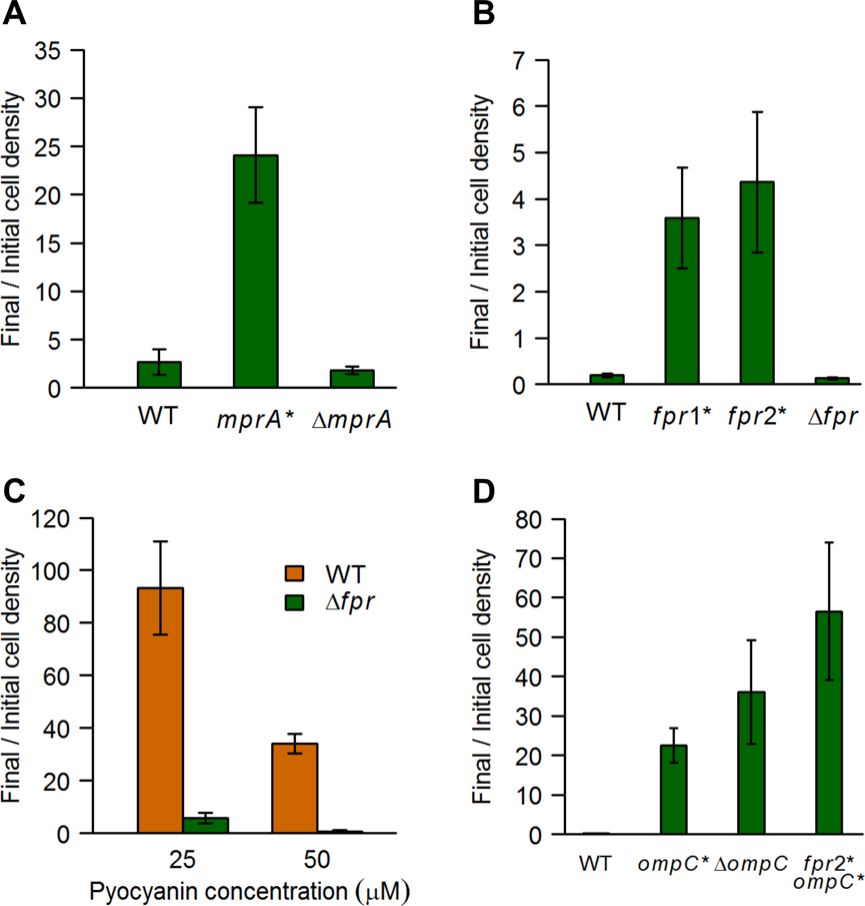
Mutations in *mprA*, *fpr*, and *ompC* increase resistance of *E*. *coli* to *P*. *aeruginosa* spent media and pyocyanin. *E*. *coli* cells were grown in the presence of either 50% (v/v) WT *P*. *aeruginosa* spent media (**A**) or 100 μM pyocyanin (**B** and **D**), or different concentrations of pyocyanin (**C**), and the cell density was determined before and after 16 hours of growth. Data are the means from at least 5 replicates. Error bars represent standard deviation in **A**, **B**, and **D**, and SEM in **C**. Mutant and WT data were compared using the one-sided Mann-Whitney *U* test followed by the Benjamini-Hochberg procedure for multiple testing correction (for **A**, **B**, and **D;** the data in parts **B** and **D** were combined for the correction). **(A)** The mprA* (but not the Δ*mprA*) data shown were significantly different from WT (*q* < 0.0005). **(B)** The fpr1* and fpr2* (but not the Δ*fpr*) data shown were significantly different from WT (*q* < 0.005). **(C)** The Δ*fpr* data were significantly different from WT (*p* < 0.005) **(D**) All mutant data shown were significantly different from WT (*q* < 0.005). Further, the fpr2* ompC* mutant data were also significantly different from the ompC* data, and the fpr2* data in part **B** (*q* < 0.005), as well as the Δ*ompC* data *(q* < 0.05).

The transcriptional repressor *mprA* negatively regulates the expression of the multidrug resistance (MDR) pump EmrAB [28], and is also predicted to regulate the MDR pump AcrAB [29]. We identified mutations in *mprA* in all sequenced clones from 5 independent populations selected against either WT *P*. *aeruginosa* or Δ*pvdJ* spent media (S1 Table). While the mutations were identical within a population, only 2 populations showed a common mutation–a single base-pair deletion at position 446 of the gene (henceforth referred to as mprA*). Thus, while there is little parallelism at the level of individual mutations, adaptive convergence is extensive at the gene level, a phenomenon seen previously in *E*. *coli* [10, 30]. All the mutations identified in *mprA* were either non-synonymous or resulted in a frameshift mutation (S1 Table) in different parts of the gene, indicative of a hypomorphic (as opposed to a hyper- or neo-morphic) phenotype. We transferred the mprA* allele to the WT parental background and tested it along with an *mprA* deletion mutant against WT *P*. *aeruginosa* spent media. Surprisingly, although the mprA* substituted strain showed significant resistance to WT *P*. *aeruginosa* spent media, the Δ*mprA* mutant did not show either increased resistance or sensitivity (Fig 8A). This suggested that either there is some compensatory regulation in the Δ*mprA* mutant, or the adaptive alleles are neomorphic, and not simple hypomorphs. The *mprA* mutants (as well as the *fpr* and *ompC* mutants described below) did not show a significant difference compared to the WT under control conditions (S5 Fig). Interestingly, *mprA* was repressed 2.5-fold in *E*. *coli* exposed to *P*. *aeruginosa* spent media supplemented with ferric citrate (in the transcriptional response measurements described above), which might be an adaptive response to exposure to antimicrobials, resulting in the upregulation of efflux pumps.

Parallel evolution at the gene level was also seen in the two independent populations selected against pyocyanin–both had mutations in the *fpr* and *ompC* genes (S1 Table). The *fpr* gene codes for the flavodoxin NADP^+^ reductase enzyme that transfers electrons between flavodoxin and NADPH, and is required for the activation of anaerobic ribonucleoside reductase, pyruvate-formate lyase and methionine synthase [31]. We identified multiple alleles of *fpr* in our strains (S1 Table), and further studied both the synonymous mutation common in population pyo1 (henceforth called fpr1*) as well as the non-synonymous mutation common in population pyo2 (henceforth called fpr2*). We transferred both alleles to the parental background, and tested them along with an *fpr* deletion strain. Interestingly, both fpr1* and fpr2*, but not Δ*fpr*, showed increased resistance to pyocyanin (Fig 8B), confirming that even the fpr1* synonymous mutation had a significant phenotypic effect. Further, the Δ*fpr* mutant showed increased sensitivity to pyocyanin at lower concentrations, compared to the WT parental strain (Fig 8C). Thus, it is likely that both the synonymous fpr1* and the non-synonymous fpr2* are hypermorphic alleles, and an increase in Fpr activity can lead to pyocyanin resistance. Pyocyanin inhibits respiration in target cells [26], which could lead to the induction of metabolic pathways that normally function under anaerobiosis, and our results suggest that Fpr activity is a limiting step for growth under these conditions. The expression of *fpr* is induced 30-fold in *E*. *coli* exposed to *P*. *aeruginosa* spent media supplemented with ferric citrate (in the transcriptional response measurements described above), which is likely a response by *E*. *coli* cells to the perceived anaerobic conditions created by pyocyanin exposure.

The *ompC* gene codes for one of the two main porins in *E*. *coli* which allow for the influx of mostly hydrophilic small molecules across the outer membrane [32]. We identified 2 different alleles of *ompC* in the 2 independent populations evolved in the presence of pyocyanin. The allele in the pyo1 population (henceforth referred to as ompC*) results in an early stop at position 54, while the allele in the pyo2 population has a frameshift that also leads to an early stop after 6 additional amino acids. Both the ompC* allele and an *ompC* deletion, in the parental background, provided significant resistance against pyocyanin, with the deletion showing approximately 2-fold higher resistance (Fig 8D), suggesting that the ompC* allele is a hypomorph. Thus, it is likely that pyocyanin enters target *E*. *coli* cells via the OmpC porin, and modulation of this protein can lead to pyocyanin resistance.

Additionally, a double mutant carrying both the ompC* and fpr2* alleles had significantly higher resistance to pyocyanin than either of the single mutants or the Δ*ompC* mutant (Fig 8D). This indicates that some pyocyanin can enter the cell even in the absence of OmpC, and increased Fpr activity can provide further resistance.

## Discussion

Our study of a *P*. *aeruginosa–E*. *coli* two-species system utilized genome-scale methods to identify the pathways and molecules that underlie all the various components of the observed *P*. *aeruginosa* antagonism against *E*. *coli*. Specific molecules that could have an effect on exogenous species under certain conditions have been identified previously, and these include siderophores [33] and phenazine molecules [25], among various others. However, here we used unbiased, agnostic methods such as HPLC and mass-spectrometry based identification of bioactive molecules, as well as measurement and computational analyses of transcriptional responses, to comprehensively characterize the specific competitive interactions present in our bi-species system under the particular conditions of our study. Such approaches can be easily carried out in less well-characterized bacterial species, thus accelerating research into the study of other basic and biomedically relevant bacterial interactions. Furthermore, the use of *E*. *coli* as a model “target” organism can also aid in the discovery of molecules underlying interspecies interactions, the immediate molecular responses elicited in target bacteria, as well as potential pathways of adaptation to such interactions.

*P*. *aeruginosa* is known to produce a wide variety of small antimicrobial molecules [8], and our results underscore the multi-pronged mode of its microbial antagonism. The combination of both interference and exploitative competition seen in a single interaction suggests that *P*. *aeruginosa* encounters other microbial species frequently in its natural habitats, and has evolved a variety of strategies to compete with this microbial diversity. In our system, sequestration of iron limits the ability of other species to carry out basic cellular processes including respiration and DNA synthesis. The target cells that are still able to grow are further exposed to phenazine molecules, which are thought to target the electron transport chain and inhibit respiration [25, 26, 34]. Lastly, the subset of target cells that are still able to carry out aerobic respiration under these conditions are then likely to be subjected to cellular toxicity due to the production of reactive oxygen radicals by the phenazine molecules [27].

Iron is a scarce resource in many environments, and competition for iron is likely to be crucial in most communities. The pyoverdine biosynthesis locus is the most divergent alignable locus in the *P*. *aeruginosa* genome [35], likely due to evolutionary pressure to counter siderophore piracy by “cheater” strains, as well as for protection against the pyocin S3 [36]. Our results suggest that it plays a role in interspecies competition as well. Regulation of pyoverdine production is dependent on iron levels, although other factors also modulate this regulation [37, 38]. Since *P*. *aeruginosa* is known to detect and respond to the presence of other species [39], and competition is thought to have shaped bacterial regulatory networks [4], it is an intriguing possibility that the induction of siderophore production may be dependent on sensing foreign species, to inhibit niche invasion.

Quorum-sensing pathways and molecules were originally thought to regulate population behavior within a species, but more recently, these molecules have been shown to have other functions, including the modulation of behaviors of exogenous species by quorum-sensing interference [40], or the regulation of antimicrobial production [41]. *P*. *aeruginosa* has multiple quorum-sensing pathways, and our results show that the PQS-pathway is important for interference competition by inducing the production of antimicrobial molecules such as the phenazines. Interestingly, while mutations in the *lasR* quorum-sensing pathway are frequently found in *P*. *aeruginosa* isolates from chronic infections [42], PQS pathway mutants have not been seen, and isolates from cystic fibrosis infections may even over-produce PQS-pathway molecules [43–45]. Thus, despite the complex interconnected quorum-sensing pathways in *P*. *aeruginosa*, there might be a separation of functions between the PQS and the classical homoserine lactone pathways, based on their roles in virulence and microbial antagonism.

While antagonistic molecules produced by various species have been identified in competitive interactions, we have limited knowledge about adaptation to such competition. Our laboratory evolution experiments identified adaptive mutations in *mprA*, *fpr* and *ompC*, demonstrating that there are multiple pathways to combat interspecies competition. Additionally, even though we evolved only a few populations in each specific condition, we still observed parallel evolution and adaptive convergence at the gene level, with multiple independent mutations in each of the above three genes, supporting the idea that these are critical genetic determinants of resistance to *P*. *aeruginosa* antimicrobials.

Laboratory evolution also revealed the OmpC porin to be a major means of pyocyanin entry into target cells, suggesting that this antimicrobial pathway takes advantage of endogenous membrane permeability routes to enter the cell. The Fpr protein is known to be important for activation of the anaerobic ribonucleoside reductase, pyruvate-formate lyase, and methionine synthase [31], and ribonucleoside reductase is the rate-limiting step for DNA synthesis [46]. The modulation of pyocyanin resistance by Fpr suggests that by inhibiting respiration [26], pyocyanin likely causes cellular metabolism to shift to anaerobic pathways, and the activation of anaerobic ribonucleoside reductase underlies the role of Fpr in acquisition of pyocyanin resistance. Mutants lacking Fpr have also been shown to have increased sensitivity to paraquat, which is a redox-cycling drug, and mutants that overproduce this protein are resistant to this drug [31], further supporting the notion that Fpr activity is rate limiting for anaerobic growth.

The MprA transcriptional repressor is known to negatively regulate the transcription of the MDR pumps [28, 29], and non-synonymous mutations in this gene have been shown to confer resistance against compounds such as thiolactomycin and CCCP [28]. Thus a loss-of-function mutation in *mprA* could likely result in resistance to the *P*. *aeruginosa* antimicrobials. However, the lack of a phenotype seen in the Δ*mprA* mutant implies that the mutations we identified in *mprA* may be neomorphic despite the fact that there are 4 different mutations found in different parts of the protein.

Two independent populations in our selections showed the same identical mprA* mutation–a deletion of a single base pair at position 446 which causes a frameshift mutation resulting in a novel 26 amino-acid C-terminus. Interestingly, an almost identical C-terminus (with a difference of only 2 amino acids between the two proteins, both of which are positive matches) was identified previously in a pathogenic isolate ECA-0157 from clinical bovine mastitis [47], raising the possibility that the adaptive pathways we identified may be relevant for interspecies interactions seen in natural niches.

Our results clearly show that even bi-species microbial interactions can be complex, including both exploitative and interference competition, and involving multiple genetic determinants and mechanisms. We provide a framework for identifying the actual fitness-determining interactions under any condition, and demonstrate the utility of applying systems-biology approaches to such problems. This framework can be expanded and applied to the study of bacterial interactions in diverse settings, including competitive and cooperative interactions within healthy and diseased states of the human microbiome, as well as polymicrobial infections. Approaches similar to those presented here can also help elucidate how stable microbial communities are formed and maintained, and how community structure can be manipulated.

## Materials and Methods

### Strains and growth conditions

All strains used in this work are described in S2 Table.

For all experiments in liquid media, bacterial strains were grown in modified M63 media [48] (13.6g/L KH_2_PO_4_, 2g/L (NH_4_)_2_SO_4_, 2μM ferric citrate, 1mM MgSO_4_; pH adjusted to 7.0 with KOH) supplemented with 0.3% glucose and 5g/L casamino acids, at 37°C, and shaken at 250rpm. For the *P*. *aeruginosa*–*E*. *coli* competition assays, the bacterial mixtures were plated on M9 + 0.5% lactose plates to select for *E*. *coli*, and on M9 + 10mM sodium citrate plates to select for *P*. *aeruginosa*. M9 media [49] contained 12.8g/L Na_2_HPO_4_.7H_2_O, 3g/L KH_2_PO_4_, 1g/L NH_4_Cl, 0.5g/L NaCl, 0.1mM CaCl_2_ and 2mM MgSO_4_. For the spent media resistance assays, cells were plated on LB plates. Strains were grown in LB liquid media (10g/L Bacto-tryptone, 5g/L yeast extract, 10g/L NaCl) or on LB plates for routine cloning and strain construction. Salt-free LB + sucrose plates contained 10g/L Bacto-tryptone, 5g/L yeast extract and 10% v/v sucrose. All plates contained 15g/L agar. The antibiotic concentrations used are listed in S3 Table.

### Construction of *P*. *aeruginosa* deletion mutants

For all bi-parental conjugations, the donor and recipient cells were grown overnight shaking at 250rpm at 37°C in LB (with the appropriate antibiotic, if required); 0.5ml of each overnight culture was used per conjugation. The overnight cultures were washed twice with PBS, and resuspended in 1/10^th^ the original volume of 100mM MgSO_4_. Multiple mating spots from a 1:1 mixture of the two parental strains were placed on LB plates, and incubated at 37°C for 3–4 hours. Cells were scraped off, collected in PBS, and plated on the appropriate selection plates.

We generated all single and multiple in-frame gene deletion mutants except for the phenazine deletion mutants (in *P*. *aeruginosa* strain PA14) using the Gateway-compatible vector pEX18ApGW [50], similar to that described in [50]. We amplified a FRT-site flanked Gentamycin resistance cassette (Gm^R^) by PCR from a pPS856 plasmid template [50]. We also amplified ~600bp fragments flanking the gene of interest by PCR (all primer sequences for the deletion constructs are listed in S4 Table), and then carried out PCR overlap extension between these 3 fragments to generate the mutant cassette. This cassette was cloned into the Gateway Entry vector PCR8/GW/TOPO (Invitrogen) by TA cloning, and transferred to the pEX18ApGW plasmid via an LR reaction using the LR Clonase II Enzyme mix (Invitrogen). The cloned fragments were verified at each stage by sequencing.

The final knockout plasmid was transformed into the conjugative S17-1 λ-pir *E*. *coli* strain, and then transferred to the parental *P*. *aeruginosa* strain using bi-parental conjugation, followed by selection on LB + irgasan + gentamicin plates. Individual conjugant colonies were streaked on salt-free LB + sucrose plates, and sucrose-resistant colonies were streaked out on LB + gentamicin and LB + carbenicillin plates. Gentamicin-resistant carbenicillin-sensitive clones were verified for the gene knockout by sequencing the target locus. To remove the gentamicin-resistance cassette, the pFLP2 plasmid expressing the Flp recombinase [51] was transferred to these knockout strains via a bi-parental conjugation with a pFLP2 carrying *E*. *coli* S17-1 λ-pir strain, followed by selection on LB + irgasan + carbenicillin plates. Individual conjugant colonies were streaked on salt-free LB + sucrose plates, and sucrose-resistant colonies were streaked out on LB, LB + gentamicin, and LB + carbenicillin plates. Gentamicin- and carbenicillin-sensitive clones were verified for proper recombination by sequencing the target locus.

The phenazine mutants were generated in strain PA14 using the pΔ*phzA1-G1* and pΔ*phzA2-G2* knockout plasmids [24]. These plasmids were transformed individually into *E*. *coli* S17-1 λ-pir, and pΔ*phzA1-G1* was mobilized into the parental strain using bi-parental conjugation, followed by selection on LB + irgasan + gentamycin plates. Individual conjugant colonies were streaked on salt free LB + sucrose plates, to resolve merodiploids, and sucrose-resistant clones were verified for the *phzA1-G1* knockout by sequencing the target locus. Subsequently, a *phzA2-G2* deletion was similarly generated in the *phzA1-G1* mutants to obtain a phenazine deletion mutant.

All single mutants (as well as the Δ*phzA1-G1* Δ*phzA2-G2* mutant) were generated in the PA14 strain. For the multiple gene knockouts, the above protocol was followed multiple times in succession for each gene deletion.

### Preparation of spent media

Overnight cultures of *P*. *aeruginosa* or *E*. *coli* strains were diluted 1:100 in fresh media, shaken at 37°C at 250rpm for 22h, and then centrifuged at 5000g for 20 minutes. The supernatant was passed through a 0.22μm filter, aliquoted if required, and stored at -20°C.

### Spent media resistance time-course

To determine the time-course of the response of *E*. *coli* to WT *P*. *aeruginosa* spent media, an overnight culture of WT *E*. *coli* was diluted 1:250 in fresh media, and grown for 1.5h shaking at 250rpm at 37°C. 500μl of this culture was added to either 500μl of spent media, 200μl spent media + 300μl of 1X M63 salts, 100μl spent media + 400μl of 1X M63 salts, or 500μl 1X M63 salts (for the control). Appropriate dilutions of the starting culture in 1X PBS were plated on LB plates, and the cultures were grown shaking at 250rpm at 37°C. Aliquots were removed from these cultures at the appropriate time-points, diluted appropriately in 1X PBS, and plated on LB plates. Samples were diluted and plated in triplicate, and the plate counts were averaged across the replicates.

The time-course of the response of *P*. *aeruginosa* to WT *E*. *coli* spent media was determined similarly–an overnight culture of WT *P*. *aeruginosa* was diluted 1:250 in fresh media, and grown for 1.5h shaking at 250rpm at 37°C. 500μl of this culture was added to either 500μl of spent media, 200μl spent media + 300μl of 1X M63 salts, or 500μl 1X M63 salts (for the control). Appropriate dilutions of the starting culture in 1X PBS were plated on LB plates, and the cultures were grown shaking at 250rpm at 37°C. Aliquots were removed from these cultures at the appropriate time-points, diluted appropriately in 1X PBS, and plated on LB plates.

### Spent media and pyocyanin resistance assays

To measure the resistance of *E*. *coli* to *P*. *aeruginosa* spent media, an overnight culture of *E*. *coli* was diluted 1:250 in fresh media, and grown for 1.5h shaking at 250rpm at 37°C. 500μl of this culture was added to either 500μl of spent media, 200μl spent media + 300μl of 1X M63 salts, 500μl 1X M63 salts + appropriate volumes of 20mM pyocyanin, or 500μl 1X M63 salts (for the control), and the cultures were grown for 16h shaking at 250rpm at 37°C. Thus, all samples had only 50% of fresh media with the rest being made up of spent media + 1X M63 salts (without glucose or casamino acids), to enable comparison between the samples. The cultures were diluted in 1X PBS and plated on LB plates before and after growth in the presence of spent media or pyocyanin to obtain the fold change in cell-density.

### Competition assays

For the *E*. *coli–P*. *aeruginosa* competitions, overnight cultures of the competing strains were diluted 1:250 in fresh media, shaken at 250rpm at 37°C for 90 minutes, and then mixed at a 1:1 ratio. Appropriate dilutions of the strains in PBS were plated on M9 + lac and M9 + citrate plates as selective conditions for *E*. *coli* and *P*. *aeruginosa* respectively. Appropriate dilutions were also plated after 20 hours of shaking at 250rpm at 37°C. Samples were diluted and plated in triplicate, and the plate counts were averaged across the replicates.

The mean cell densities for each competitor were used to calculate the effective growth rate *m* (the realized Malthusian parameter) as the number of doublings over the duration of the competition [52, 53]:

$$m_{\mathrm{Strain}}\;={\text{log}}_{2}\;(N_{f}/N_{i})\;/\;\text{t}$$

where *N*_*i*_ and *N*_*f*_ are the initial and final cell densities, and *t* is the duration of the competition. The relative fitness of strain A to its competitor strain B was then calculated as the ratio of their effective growth rates (*m*_*A*_ / *m*_*B*_).

### Transcriptional profiling

To measure the transcriptional response of *E*. *coli* to *P*. *aeruginosa* spent media, we diluted an overnight culture of the *E*. *coli* MG1655 into 40ml of media, to a final A_600_ of 0.05 (~130-fold dilution). The cultures were incubated shaking at 250rpm at 37°C for 2 hours. We added 10ml of WT *P*. *aeruginosa* spent media to the flask, and immediately removed 2.5ml of the mixture for the 0 minute time-point. Subsequently, we removed a similar aliquot after 20 minutes of shaking at 250rpm at 37°C. Two replicates were performed for this experiment.

We added each aliquot immediately to 5ml of the RNAprotect Bacteria Reagent (Qiagen), incubated at room temperature for 5 minutes, and then centrifuged at 5000g for 10 minutes. We discarded the supernatant, and stored the pellets at -80°C. We isolated RNA from these samples using the Total RNA Purification Kit (Norgen), as per the manufacturer’s protocol for bacteria.

To label the RNA, we first polyadenlyated it, by combining 25μl of the undiluted RNA with 5μl 10X Poly(A) Polymerase Reaction Buffer (New England Biolabs), 5μl 10 mM ATP, and 1μl (5 U) *E*. *coli* Poly(A) polymerase (New England Biolabs) in a total volume of 50μl, and incubating at 37°C for 30 minutes, followed by a 20 minute incubation at 65°C to inactivate the enzyme. We cleaned the samples using the RNeasy Mini Kit from Qiagen, and then labeled 300ng of the 0 minute RNA with cyanine 3-CTP, and 300ng of the 20 minute sample with cyanine 5-CTP using the Low Input Quick Amp Labeling Kit (Agilent). We hybridized the two samples on custom tiling arrays from Agilent (Design ID 024568) [52], according to the manufacturer’s protocol.

To measure the transcriptional response of *E*. *coli* to *P*. *aeruginosa* spent media in the presence of iron, we diluted overnight cultures of the *E*. *coli* MG1655 into 24.75ml of media, to a final A_600_ of 0.05 (~130-fold dilution). The cultures were incubated shaking at 250rpm at 37°C for 105 minutes. We added 20.25ml of WT *P*. *aeruginosa* spent media and 100μM ferric citrate to the flask, and immediately removed 2ml of the mixture for the 0 minute time-point. Subsequently, we removed a similar aliquot after 20 minutes of shaking at 250rpm at 37°C, and processed the aliquots as above. Two replicates were performed for the experiment.

### Analysis of microarray data

The fluorescence intensities were extracted using the Agilent Feature Extraction Software Version 9.5, using the protocol GE2-v5_95_Feb07 without spike-in controls. The probes were filtered using the IsFound, IsFeatNonUnif, IsBGNonUnif, ISFeatPopnOL, and IsBGPopnOL flags, and discarded if the first flag had a value of 0, or any of the others had a value of 1. We used the ‘LogRatio’ value for each probe, and all probes which were on the sense strand of the coding region of a gene were assigned to the gene. The values were averaged across all probes for a gene, and across the two biological replicates for each experiment.

We ran iPAGE [13] in continuous mode with various numbers of bins, which did not significantly change the categories identified. The ‘GO annotation’ module was used for the data shown in Fig 2, and the ‘Transcription factor regulon’ module was used for the data shown in Fig 6.

### Mass-spectrometry analysis of spent media

Whole spent media from WT *P*. *aeruginosa* was analyzed using both MALDI-TOF and ESI static nanospray mass spectrometry. The main component seen in the spent media was the same in both spectra, and had a mass of 1335 Daltons. The mass spectrometry analysis was performed at the Protein Core Facility at Columbia University.

For the analysis of active fractions of *P*. *aeruginosa* spent media, spent media from WT *P*. *aeruginosa* was fractionated by HPLC-MS using a C_18_ reverse-phase column in a linear 5%–95% acetonitrile-water gradient, with a flow-rate of 1 ml/minute for 90 minutes. Fractions were collected every 2 minutes for a total of 45 fractions. The fractions were dried using a Savant DNA120 concentrator and resuspended in 200μl water. The growth of *E*. *coli* cells was then tested against 20% (v/v) of the resuspended fractions in 100μl media in a 96-well plate, starting from a 1:100 dilution of an overnight culture of *E*. *coli*. A sample with no spent media fractions was used as the control. The media was covered with 100μl mineral oil to prevent evaporation. The plate was shaken continuously without the lid at the ‘medium’ setting at 37°C for 22 hours in a Biotek Synergy MX plate reader. The absorbance at 600nm was read, and fractions which inhibited the fold-change in absorbance more than 10-fold compared to the control were identified. Three consecutive active fractions had a mass-spectrometry profile with the same single peak (shown in S2 Fig). The HPLC-MS was performed at the Princeton Proteomics and Mass Spectrometry Core Facility.

### Laboratory evolution of spent media and pyocyanin resistance

WT *E*. *coli* cells were grown shaking at 250 rpm at 37°C, in the presence of increasing concentrations of the selective agent (WT *P*. *aeruginosa* spent media, Δ*pvdJ* spent media, or pyocyanin), with a daily 50–100 fold dilution into 1 ml fresh media in snap-cap tubes containing the selective agent. 7 daily transfers were carried out for the WT *P*. *aeruginosa* spent media (concentration increasing from 12.5–35% (v/v)), 15 for the Δ*pvdJ* spent media (concentration increasing from 30–70% (v/v)), and 19 for pyocyanin (concentration increasing from 75–800 μM). Following the selections, 2 populations each evolved in the presence of WT spent media and pyocyanin, and 3 populations evolved in the presence of Δ*pvdJ* spent media were streaked out to obtain individual clones and 2–3 individual clones were analyzed by whole-genome sequencing.

### Whole-genome sequencing of evolved strains

We prepared genomic DNA from the evolved clones using the Qiagen DNeasy Blood and Tissue kit, and prepared indexed paired-end libraries from the DNA using the Illumina Nextera XT DNA Library Preparation kit. The samples were pooled and sequenced on a NextSeq 500 (Illumina) for 150 cycles. The bcl2fastq package from Illumina was used to demultiplex the data and obtain FASTQ files for each sample. The Illumina adapters were removed using *cutadapt* [54] and the sequences were trimmed to remove poor quality bases at the ends using *trimmomatic* [55]. The sequences from each sample were then analyzed using the default settings of *breseq-0*.*26* [56], to identify the mutations in the evolved strain compared to the parental MG1655 background. The samples had an average of 20–45x coverage over the genome. The *breseq-0*.*26* pipeline identifies any variants between the given sequence and a reference genome (in this case Genbank Accession NC_000913.2). We only focused on the high-confidence mutations, and do not report the marginal predictions. The parental strain used also has mutations compared to the reference genome (listed in S5 Table), some of which have been previously reported [57]. The mutations identified in the evolved clones compared to the ancestral genome are listed in S1 Table.

### Transfer of mutant alleles from evolved strains to WT *E*. *coli*

We generated all single and multiple allele-replacements (in the parental *E*. *coli* MG1655 strain) using the pKOV plasmid [58, 59]. We amplified the evolved allele from the appropriate strain, including ~500bp flanking the mutation on both sides, using primers that had 20–25bp overlap with the ends of the pKOV plasmid linearized with BamHI/NotI (all primer sequences for the constructs are listed in S6 Table). The pKOV plasmid was digested with BamHI and NotI (New England Biolabs) and the 5.6kb fragment was purified using the Zymoclean Gel DNA Recovery Kit. The mutant allele was then cloned into pKOV using Gibson Assembly, and the cloned fragment verified by sequencing.

The allele replacement was carried out similar to the original protocol [59]. The allele-replacement plasmid was transformed into the appropriate strain, followed by selection on LB + chloramphenicol plates at 42°C to obtain integrants. Individual colonies were re-streaked out on LB + chloramphenicol plates at 42°C to reduce the background of non-integrants. Chloramphenicol-resistant clones were streaked out on salt-free LB + sucrose plates at 30°C to resolve the integration and individual sucrose-resistant colonies were tested for the allele-replacement by PCR with mismatched primers [60]. Strains with the appropriate allele replacement were streaked out on LB + chloramphenicol plates at 30°C and chloramphenicol sensitive clones were verified for the allele replacement by sequencing the target locus.

All single mutants were generated in the WT *E*. *coli* strain. For the multiple allele replacements, the above protocol was followed multiple times in succession for each allele.

### Construction of *E*. *coli* deletion mutants

The single gene deletions were obtained from the Keio collection [61] and transferred to the WT *E*. *coli* MG1655 background using P1 vir transduction [49], followed by selection on LB + Kanamycin plates. Kanamycin-resistant clones were tested for the appropriate mutation by PCR, and then cured of the kanamycin resistance cassette by transforming with the plasmid pcp20 [62], and selecting on LB + Ampicillin plates at 30°C. Ampicillin resistant clones were streaked out on LB plates and incubated at 42°C for 24 hours, and then streaked out on LB, LB + Ampicillin and LB + Kanamycin plates. Ampicillin- and kanamycin-sensitive clones were verified for the deletion by sequencing the target locus.

### Data availability

The microarray data have been deposited in the Gene Expression Omnibus (GEO) with the accession number GSE72283. The whole genome sequencing data have been deposited in the Sequence Read Archive, associated with the BioProject PRJNA292975.

## Supporting Information

S1 Fig *E*. *coli* spent media does not affect *P*. *aeruginosa* growth. *P*. *aeruginosa* cells were grown in the presence of different % (v/v) of WT *E*. *coli* spent media, and the cell density was determined at several time-points. Data are the means from 5 replicates, and the error bars show the standard deviation. None of the spent media data (at 22h) were significantly different from the control (*q* > 0.1) as determined by a two-sided Mann-Whitney *U* test followed by the Benjamini-Hochberg procedure for multiple testing correction.
(TIF)

S2 Fig The main component of WT *P*. *aeruginosa* spent media has an *m/*z which is identical to the molecular mass of pyoverdine. *P*. *aeruginosa* was subjected to MALDI mass spectrometry using an HCCA matrix. The spectrum depicted in the figure shows that the main component in the spent media has an *m/z* of 1335 Daltons, which matches the molecular mass of the main *P*. *aeruginosa* siderophore pyoverdine.
(TIF)

S3 Fig An active fraction of WT *P*. *aeruginosa* spent media consists of a molecule whose *m/z* matches the mass of pyoverdine. *P*. *aeruginosa* spent media was fractionated and analyzed by HPLC-MS, and the fractions were tested for growth-inhibitory activity against *E*. *coli*. The mass-spectrometry profile shown in the figure is that of the three fractions collected between 10–16 minutes (all three fractions showed growth-inhibitory activity against *E*. *coli*). The *m/z* of the main component (1335 Daltons) matches the molecular mass of the siderophore pyoverdine.
(TIF)

S4 Fig Supplementation of iron causes a marginal increase in growth under control conditions. *E*. *coli* WT cells were grown in the presence of 50% media salts base supplemented with 0, 10 and 100 μM ferric citrate, and the cell density was determined before and after 16 hours of growth. Data are the means from 5 replicates. Error bars represent standard deviation. Both 10 and 100 μM ferric citrate supplementation data were significantly different from the control (*q* < 0.05), but not from each other (*q* > 0.3), as determined by a two-sided Mann-Whitney *U* test followed by the Benjamini-Hochberg procedure for multiple testing correction.
(TIF)

S5 Fig *E*. *coli* allele-replacement and deletion mutants are similar to WT under control conditions. *E*. *coli* WT and mutant cells were grown in the presence of 50% media salts base, and the cell density was determined before and after 16 hours of growth. Data are the means from at least 5 replicates. Error bars represent standard deviation. None of the mutant data shown were significantly different from WT (*q* > 0.1) as determined by a two-sided Mann-Whitney *U* test followed by the Benjamini-Hochberg procedure for multiple testing correction. The Δ*fpr* and Δ*mprA* mutants had a *p*-value of 0.03175 prior to the multiple testing correction, suggesting that their lesser growth compared to WT under control conditions may be marginally significant.
(TIF)

S1 Table Mutations identified in the evolved strains compared to the parental MG1655. (PDF)

S2 Table Bacterial strains used in this study. (PDF)

S3 Table Antibiotic concentrations used during strain construction. (PDF)

S4 Table Sequences of primers used for the construction of the *P*. *aeruginosa* mutant strains. (PDF)

S5 Table Mutations identified in the parental MG1655 strain compared to the reference sequence. (PDF)

S6 Table Sequences of primers used for the construction of the *E*. *coli* allele-replacement strains. (PDF)
