# Supplementary figures and images for: Multifactorial Competition and Resistance in a Two-Species Bacterial System
Source: PLoS Genet. 2015 Dec 8;11(12):e1005715. doi: 10.1371/journal.pgen.1005715 (PMC4672897; doi:10.1371/journal.pgen.1005715)

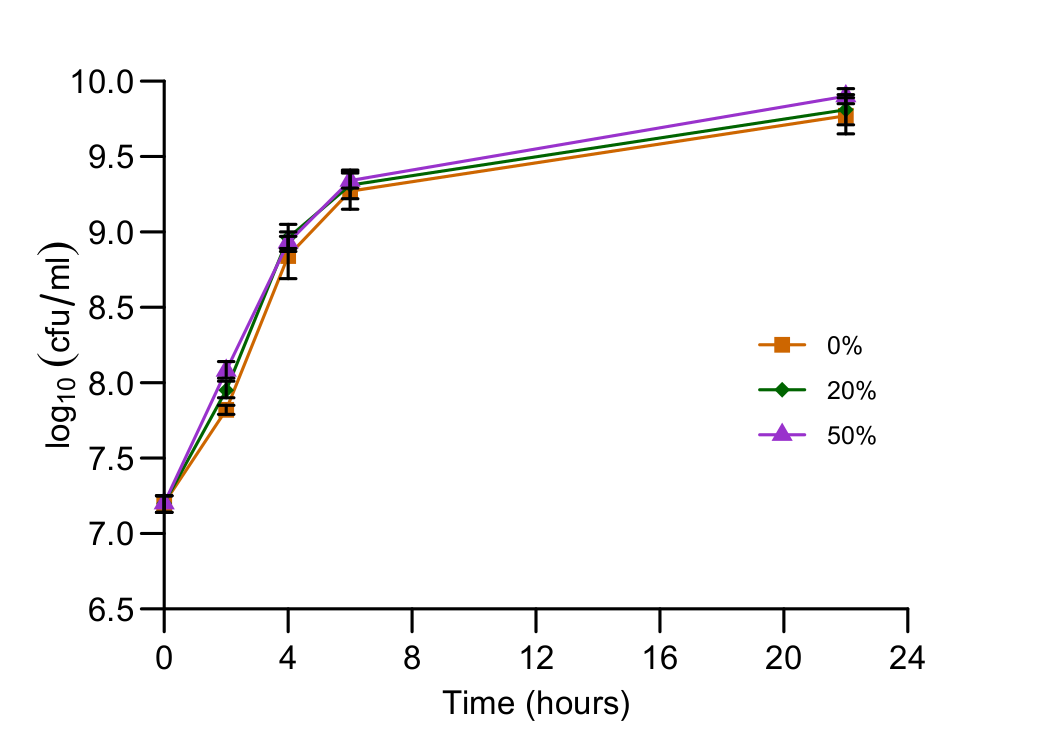

Supplement: S1 Fig — P. aeruginosa cells were grown in the presence of different % (v/v) of WT E. coli spent media, and the cell density was determined at several time-points. Data are the means from 5 replicates, and the error bars show the standard deviation. None of the spent media data (at 22h) were significantly different from the control (q > 0.1) as determined by a two-sided Mann-Whitney U test followed by the Benjamini-Hochberg procedure for multiple testing correction. (TIF) [file pgen.1005715.s001.tif]

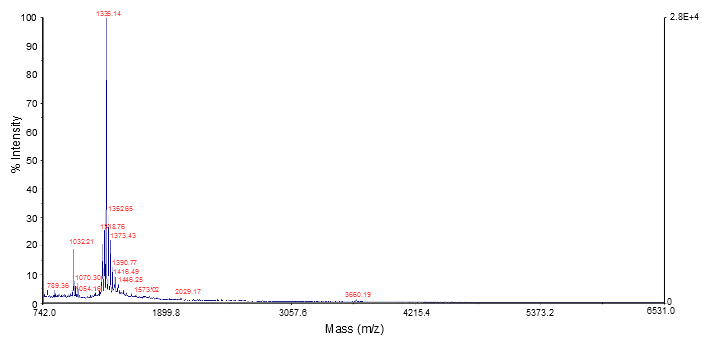

Supplement: S2 Fig — P. aeruginosa was subjected to MALDI mass spectrometry using an HCCA matrix. The spectrum depicted in the figure shows that the main component in the spent media has an m/z of 1335 Daltons, which matches the molecular mass of the main P. aeruginosa siderophore pyoverdine. (TIF) [file pgen.1005715.s002.tif]
