# Supplementary material for: Multifactorial Competition and Resistance in a Two-Species Bacterial System
Source: PLoS Genet. 2015 Dec 8;11(12):e1005715. doi: 10.1371/journal.pgen.1005715 (PMC4672897; doi:10.1371/journal.pgen.1005715)
Supplement: S1 Table — (PDF) [file pgen.1005715.s006.pdf]

S1 Table. Mutations identified in the evolved strains compared to the parental MG1655.

Selective Agent	Population	Strain	Position	Mutation	Annotation	Gene
WT spent media	WT2	WT2_2	611,871	G→T	intergenic (-154/-167)	*fepA / fes*
			903,861	G→T	E16* (GAA→TAA)	*ybjQ*
			1,195,443	C→T	H366H (CAC→CAT)	*icd*
			1,195,455	C→T	T370T (ACC→ACT)	*icd*
			1,195,468	T→C	L375L (TTA→CTA)	*icd*
			1,195,470	A→G	L375L (TTA→TTG)	*icd*
			1,982,212	C→G	G294G (GGG→GGC)	*araG*
			2,808,978	A→C	T63P (ACG→CCG)	*mprA*
			4,275,901	A→G	D137G (GAC→GGC)	*soxR*
		WT2_3	611871	G→T	intergenic (-154/-167)	*fepA / fes*
			2808978	A→C	T63P (ACG→CCG)	*mprA*
			3578318	Δ3,133 bp	IS1-mediated	*[yhhX]–yrhA*
			4294404	+GC	intergenic (+587/+55)	*gltP / yjcO*
	WT3	WT3_2	1,208,643	C→G	pseudogene (200/501 nt)	*stfE*
			2,150,268	C→G	E77Q (GAA→CAA)	*yegK*
			2,809,057	C→T	T89M (ACG→ATG)	*mprA*
			3,577,701	Δ3,750 bp	IS*1*-mediated	*yhhX–yrhA*
		WT3_3	2,809,057	C→T	T89M (ACG→ATG)	*mprA*
			3,577,701	Δ3,750 bp	IS*1*-mediated	*yhhX–yrhA*
Δ*pvdJ* spent media	pvdJ1	pvdJ1_2	**2,809,237**	**Δ1 bp**	**coding (446/531 nt)**	***mprA***
		pvdJ1_3	**2,809,237**	**Δ1 bp**	**coding (446/531 nt)**	***mprA***
	pvdJ2	pvdJ2_1	1,208,643	C→G	pseudogene (200/501 nt)	*stfE*
			**2,809,237**	**Δ1 bp**	**coding (446/531 nt)**	***mprA***
			4,294,404	+GC	intergenic (+587/+55)	*gltP / yjcO*
		pvdJ2	**2,809,237**	**Δ1 bp**	**coding (446/531 nt)**	***mprA***

		_2				
			3,577,292	Δ4,159 bp	IS*1*-mediated	*[yhhW]–yrhA*
			4,294,404	+GC	intergenic (+587/+55)	*gltP / yjcO*
		pvdJ2_3	1,040,609	G→C	G257A (GGA→GCA)	*appA*
			**2,809,237**	**Δ1 bp**	**coding (446/531 nt)**	***mprA***
			3,578,830	Δ2,622 bp	IS*1*-mediated	*yhhY–yrhA*
	pvdJ3	pvdJ3_1	485,346	IS*1* (–) +8 bp	coding (362-369/648 nt)	*acrR*
			2,809,117	A→C	D109A (GAT→GCT)	*mprA*
		pvdJ3_2	485,346	IS*1* (–) +8 bp	coding (362-369/648 nt)	*acrR*
			1,525,626	G→C	intergenic (+450/-300)	*yncH / rhsE*
			2,809,117	A→C	D109A (GAT→GCT)	*mprA*
			4,205,587	A→C	intergenic (-32/-583)	*purH / rrsE*
		pvdJ3_3	2,809,117	A→C	D109A (GAT→GCT)	*mprA*
			3,583,704	G→T	G381G (GGC→GGA)	*ggt*
			3,726,206	C→G	A397P (GCG→CCG)	*xylB*
			4,176,250	T→G	V162G (GTT→GGT)	*nusG*
			4,205,587	A→C	intergenic (-32/-583)	*purH / rrsE*
			4,294,404	+GC	intergenic (+587/+55)	*gltP / yjcO*
pyocyanin	pyo1	pyo1_1	**2,310,612**	**G→A**	**Q54* (CAG→TAG)**	***ompC***
			**4,112,442**	**G→A**	**A18A (GCC→GCT)**	***fpr***
			4,112,479	G→A	T6I (ACA→ATA)	*fpr*
		pyo1_2	1,040,609	G→C	G257A (GGA→GCA)	*appA*
			1,754,820	G→T	E367* (GAA→TAA)	*pykF*
			1,755,411	C→G	intergenic (+277/-34)	*pykF / lpp*
			**2,310,612**	**G→A**	**Q54* (CAG→TAG)**	***ompC***
			3,309,402	IS*4* (+) +12 bp	intergenic (-212/+24)	*pnp / rpsO*
			**4,112,442**	**G→A**	**A18A (GCC→GCT)**	***fpr***
		pyo1_3	1,754,820	G→T	E367* (GAA→TAA)	*pykF*
			1,755,411	C→G	intergenic (+277/-34)	*pykF / lpp*

			**2,310,612**	**G→A**	**Q54* (CAG→TAG)**	***ompC***
			**4,112,442**	**G→A**	**A18A (GCC→GCT)**	***fpr***
	pyo2	pyo2_1	729,877	T→C	C358R (TGT→CGT)	*rhsC*
			2,310,234	+TC	coding (538/1104 nt)	*ompC*
			**4,112,492**	**C→A**	**A2S (GCT→TCT)**	***fpr***
		pyo2_2	1,525,626	G→C	intergenic (+450/-300)	*yncH / rhsE*
			2,310,234	+TC	coding (538/1104 nt)	*ompC*
			**4,112,492**	**C→A**	**A2S (GCT→TCT)**	***fpr***
		pyo2_3	729,877	T→C	C358R (TGT→CGT)	*rhsC*
			2,310,234	+TC	coding (538/1104 nt)	*ompC*
			**4,112,492**	**C→A**	**A2S (GCT→TCT)**	***fpr***
			4,294,403	+G	intergenic (+586/+56)	*gltP / yjcO*

The mutations in **bold** were transferred to the parental WT strain and tested further.
