# Supplementary material for: Multifactorial Competition and Resistance in a Two-Species Bacterial System
Source: PLoS Genet. 2015 Dec 8;11(12):e1005715. doi: 10.1371/journal.pgen.1005715 (PMC4672897; doi:10.1371/journal.pgen.1005715)
Supplement: S2 Table — (PDF) [file pgen.1005715.s007.pdf]

S2 Table. Bacterial Strains used in this study.

Species	Genotype	Source
E. coli	MG1655	ATCC 47076
	*fpr1**	This study
	*fpr2**	This study
	*mprA**	This study
	*ompC**	This study
	*ompC** *fpr2**	This study
	Δ*fpr*	This study
	Δ*mprA*	This study
	Δ*ompC*	This study
P. aeruginosa	PA14	[1]
	Δ*pvdJ*	This study
	Δ*pchE*	This study
	Δ*pqsA*	This study
	Δ*pqsE*	This study
	Δ*phzA1-G1* Δ*phzA2-G2*	This study
	Δ*pvdJ* Δ*pchE*	This study
	Δ*pvdJ* Δ*pchE* Δ*pqsA*	This study
	Δ*pvdJ* Δ*pchE* Δ*pqsE*	This study
	Δ*pvdJ* Δ*pchE* Δ*phzA1-G1* Δ*phzA2-G2*	This study

1. Rahme LG, Stevens EJ, Wolfort SF, Shao J, Tompkins RG, Ausubel FM. Common virulence factors for bacterial pathogenicity in plants and animals. Science. 1995;268(5219):1899-902. doi: 10.1126/science.7604262.
