# Supplementary material for: Multifactorial Competition and Resistance in a Two-Species Bacterial System
Source: PLoS Genet. 2015 Dec 8;11(12):e1005715. doi: 10.1371/journal.pgen.1005715 (PMC4672897; doi:10.1371/journal.pgen.1005715)
Supplement: S3 Table — (PDF) [file pgen.1005715.s008.pdf]

S3 Table. Antibiotic concentrations used during strain construction.

**Antibiotic**	**Concentration**
Ampicillin	100 µg/ml
Spectinomycin	100 µg/ml
Carbenicillin	200 µg/ml
Gentamycin	50 µg/ml
Irgasan	25 µg/ml
Chloramphenicol	30 µg/ml
