# Supplementary material for: Multifactorial Competition and Resistance in a Two-Species Bacterial System
Source: PLoS Genet. 2015 Dec 8;11(12):e1005715. doi: 10.1371/journal.pgen.1005715 (PMC4672897; doi:10.1371/journal.pgen.1005715)
Supplement: S4 Table — (PDF) [file pgen.1005715.s009.pdf]

S4 Table. Sequences of primers used for the construction of the *P. aeruginosa* mutant strains.

**Primer Description**	**Primer sequence**[a]
Forward primer for FRT-flanked gentamycin-resistance cassette	CGA ATT AGC TTC AAA AGC GCT CTG A
Reverse primer for FRT-flanked gentamycin-resistance cassette	CGA ATT GGG GAT CTT GAA GTT CCT
Forward primer for upstream fragment of *pvdJ*	GAG GCT TCC GCA TCG AAC TG
Reverse primer for upstream fragment of *pvdJ*	**TCAGAGCGCTTTTGAAGCTAATTCG** CAG GTT TGG ATT GGA AAG CAT G
Forward primer for downstream fragment of *pvdJ*	**AGGAACTTCAAGATCCCCAATTCG** GCG CTC AAA CGT CTA TCT G
Reverse primer for downstream fragment of *pvdJ*	GCT CGA GCC CTC GAC TAT AG
Forward primer for upstream fragment of *pchE*	CAG CGC CCA GTT CCT TTC C
Reverse primer for upstream fragment of *pchE*	**TCAGAGCGCTTTTGAAGCTAATTCG** AGG GCG GTA CGG GAA TCG G
Forward primer for downstream fragment of *pchE*	**AGGAACTTCAAGATCCCCAATTCG ATC** GAG GAA ACC CAA CTG GAG
Reverse primer for downstream fragment of *pchE*	CCG GCC GAT ACT CAG CTC
Forward primer for upstream fragment of *pqsA*	GGC ATG TAG GTG TCC TCT TCG
Reverse primer for upstream fragment of *pqsA*	**TCAGAGCGCTTTTGAAGCTAATTCG** GCG GAA CAG AAC CTC GGT CAG
Forward primer for downstream fragment of *pqsA*	**AGGAACTTCAAGATCCCCAATTCG** GAG GAA CGG GCA TGT TGA TTC AG
Reverse primer for downstream fragment of *pqsA*	CCG GCA GGT TGA GGT GTC C
Forward primer for upstream fragment of *pqsE*	GGC ATG TCC TGG TGG TCT GC
Reverse primer for upstream fragment of *pqsE*	**TCAGAGCGCTTTTGAAGCTAATTCG** GGG AGC CGA AAG CCT CAA C
Forward primer for downstream fragment of *pqsE*	**AGGAACTTCAAGATCCCCAATTCG** CAG GCG CTG CCT CTG GAC
Reverse primer for downstream fragment of *pqsE*	GCC AGG TCG AAA CTG AAC AGG

a The sequence in **bold** overlaps with the FRT-flanked gentamycin-resistance cassette, and is required for the overlap extension PCR.
