# Supplementary material for: Multifactorial Competition and Resistance in a Two-Species Bacterial System
Source: PLoS Genet. 2015 Dec 8;11(12):e1005715. doi: 10.1371/journal.pgen.1005715 (PMC4672897; doi:10.1371/journal.pgen.1005715)
Supplement: S5 Table — (PDF) [file pgen.1005715.s010.pdf]

S5 Table. Mutations identified in the parental MG1655 strain compared to the reference sequence.

Position	Mutation	Annotation	Gene
263,322	G→T	L50L (CTC→CTA)	*ykfH* ←
547,694	A→G	pseudogene (114/252 nt)	*ylbE* →
547,835	+G	pseudogene (4/1008 nt)	*ylbE* →
1,196,725	C→T	G11R (GGA→AGA)	*ymfD* ←
1,976,527	Δ776 bp		*insB1–insA*
2,677,547	G→T	A1074D (GCT→GAT)	*yphG* ←
3,411,593	T→C	intergenic (-106/-293)	*envR* ← / → *acrE*
3,957,957	C→T	intergenic (-121/+78)	*ppiC* ← / ← *yifN*
