# Supplementary material for: Multifactorial Competition and Resistance in a Two-Species Bacterial System
Source: PLoS Genet. 2015 Dec 8;11(12):e1005715. doi: 10.1371/journal.pgen.1005715 (PMC4672897; doi:10.1371/journal.pgen.1005715)
Supplement: S6 Table — (PDF) [file pgen.1005715.s011.pdf]

S5 Table. Sequences of primers used for the construction of the *E. coli* allele-replacement strains.

**Primer Description**	**Primer sequence**[a]
Forward primer for *fpr*	**TCGGTACCCGGGGATCGC** AGC GAA GTT GAC GTG CTG
Reverse primer for *fpr*	**CCGGTCGACTCTAGAG** CGG TAT CCG TCC GGT GAG
Forward primer for *mprA*	**TCGGTACCCGGGGATCGCGGCCGC** GCG GTA GGT ATT TTG CTC
Reverse primer for *mprA*	**CCGGTCGACTCTAGAGGATCC** TCT GTC GGG TCG AGA GTG
Forward primer for *ompC*	**TCGGTACCCGGGGATCGCGGCCGC** CGG GAG TTA TTC TAG TTG C
Reverse primer for *ompC*	**CCGGTCGACTCTAGAGGATCC** TTG TCG TCC AGC AGG TTG

a The sequence in **bold** overlaps with the pKOV plasmid, and is required for the Gibson Assembly.
